# Emotional intelligence and its impact on the mental health of Chinese medical students: a questionnaire study

**DOI:** 10.1186/s12909-026-08565-5

**Published:** 2026-01-12

**Authors:** Zhonghang Xu, Fenglin Chen, Wei Li, Zhongxin Xu, Xiaohua Shi

**Affiliations:** 1 China-Japan Union Hospital of Jilin University, No. 126, Xiantai Street, Changchun, 130033 Jilin Province China; 2 Jilin Provincial Clinical Medicine Research Center of Behavioral Neurology, Changchun, Jilin Province China

**Keywords:** Emotional intelligence, Medical students, Questionnaire, Mental health

## Abstract

**Background:**

Emotional intelligence (EI) is increasingly recognized as an essential competency in medical education, yet evidence on its determinants and its relationship with mental health among Chinese medical students remains limited. This study examined factors associated with EI, explored its associations with stress, anxiety, and depression, and assessed whether EI mediates gender differences in psychological outcomes.

**Methods:**

The study involved undergraduate medical students from the 1st, 3rd, 5th, 7th, and 9th semesters at the Norman Bethune Health Science Center of Jilin University. An online questionnaire was administered, including sociodemographic questions and validated scales to assess perceived stress, anxiety, depression, and EI. Additional data on factors such as leadership experience and participation in competitive activities were also collected. Descriptive statistics, t-tests, ANOVA, Pearson correlations, and multivariate logistic regression analyses were used to evaluate associations. Mediation analyses tested whether EI mediated the relationship between gender and mental health indicators.

**Results:**

A total of 1,376 medical students were included (mean EI = 4.86 ± 0.81). EI differed significantly by gender, academic semester, leadership experience, and competition participation (all *p* < 0.05). Students reporting stress (53%), anxiety (9%), or depression (18%) had markedly lower EI scores (all *p* < 0.001). Higher EI was independently associated with lower odds of stress (OR 0.20), anxiety (OR 0.14), and depression (OR 0.12) in adjusted models (all *p* < 0.001). Mediation analysis indicated that EI partially accounted for gender differences in psychological outcomes, explaining 36.4% of the total effect on anxiety and 38.3% on depression. These findings highlight the protective role of EI in medical students’ mental health and its contribution to gender-related differences in psychological well-being.

**Conclusions:**

EI plays a protective role in the mental health of medical students and partially explains gender disparities in anxiety and depression. As EI is a modifiable competency, incorporating EI-focused training, leadership development, and experiential learning into medical curricula may strengthen students’ emotional skills and psychological resilience. Future longitudinal and multi-institutional studies are needed to evaluate the long-term impact of EI-enhancing strategies within medical education.

## Background

Emotional Intelligence (EI), first conceptualized by Salovey and Mayer (1990) as a form of cognitive-emotional ability, refers to the capacity to accurately perceive, understand, regulate, and utilize emotions in oneself and others [1]. EI is generally structured into several core components—emotional perception, emotional understanding, emotion regulation, and emotional utilization [2, 3]. These abilities together enable individuals to manage interpersonal relationships, maintain psychological balance, and adapt effectively to environmental stressors [4–7]. Trait-based models further conceptualize EI as a constellation of emotional self-perceptions, highlighting stable personality-linked dispositions that influence stress reactivity and coping [8].

EI is a fundamental competency in medical education, underpinning students’ academic performance, clinical skills, and psychological well-being. Grounded in emotion-regulation and stress–coping theories [9], EI operates as a protective mechanism: by enhancing awareness, appraisal, and regulation of emotions [10], it promotes adaptive coping strategies that mitigate the impact of psychological distress and preserve psychological resilience. Conversely, deficits in EI compromise emotion regulation and interpersonal functioning, increasing susceptibility to anxiety and depressive symptoms. Empirical studies show that medical students with higher EI manage stress more effectively, communicate more competently with patients, and form more constructive relationships with mentors and peers, thereby facilitating professional development and improving both academic and clinical outcomes [11–14]. In addition, heightened EI supports self-regulation and empathy while reducing emotional exhaustion in demanding educational and clinical environments, ultimately strengthening readiness for practice and long-term professional sustainability [15, 16].

Medical training, while essential for professional development, often exposes students to substantial psychological distress, including stress, anxiety, and depression [17, 18]. The demanding academic workload, coupled with intensive clinical placements, often elevates stress levels and disrupts the balance between personal life and studies, negatively impacting both academic performance and clinical competence. Anxiety is particularly prevalent, with medical students facing added pressures from high academic expectations, the development of clinical skills, and the competitive nature of employment and postgraduate opportunities. These stressors not only affect academic outcomes but also increase the risk of burnout. Depression is another significant concern, exacerbated by the constant demands of medical education, heavy workloads, and insufficient psychological support. These interconnected challenges threaten students’ well-being and professional growth, underscoring the need for effective interventions [19].

Particularly for Chinese medical students, they face distinct mental health challenges due to unique socio-cultural factors, the educational system, and academic pressures [20]. Additionally, there is a significant shortage of targeted, personalized psychological counseling services, compounded by the large student population, leading to limited mental health resources. Consequently, many students do not receive timely or effective support when facing psychological difficulties [21]. Therefore, it is crucial to understand the mental health status of Chinese medical students and develop tailored interventions to address their specific needs.

Research has indicated that several factors, including gender, age, semester, hospital internship time, competition activity frequency, and leadership experience, influenceEI among medical students [22]. Gender is a consistent predictor, with female students often showing higher EI, possibly due to differences in emotional expression and socialization [23]. Advancing age and academic progression are also linked to greater EI through increased maturity, clinical exposure, and reflective learning. Clinical internships and participation in competitions foster empathy, resilience, and teamwork, while leadership experience further enhances motivation, interpersonal skills, and self-management—qualities essential for effective medical practice. Collectively, these variables were selected to represent the major demographic, educational, and experiential dimensions that shape EI, thereby informing targeted curriculum strategies for its systematic development.

Gender differences represent an important but underexplored dimension in the study of EI and mental health among medical students. Prior research suggests that female students often exhibit higher EI levels than males, particularly in emotional perception and empathy [23–25]. However, findings are inconsistent, and some studies have reported comparable or even higher EI among males [22]. Similarly, gender disparities exist in mental health outcomes—female students frequently report higher stress, anxiety, and depression [18, 26], whereas male students may show greater burnout and emotional detachment. These differences may arise from sociocultural expectations, emotional expression norms, and biological stress responses, but the role of EI in mediating these gender effects remains insufficiently understood.

**Fig. 1:**
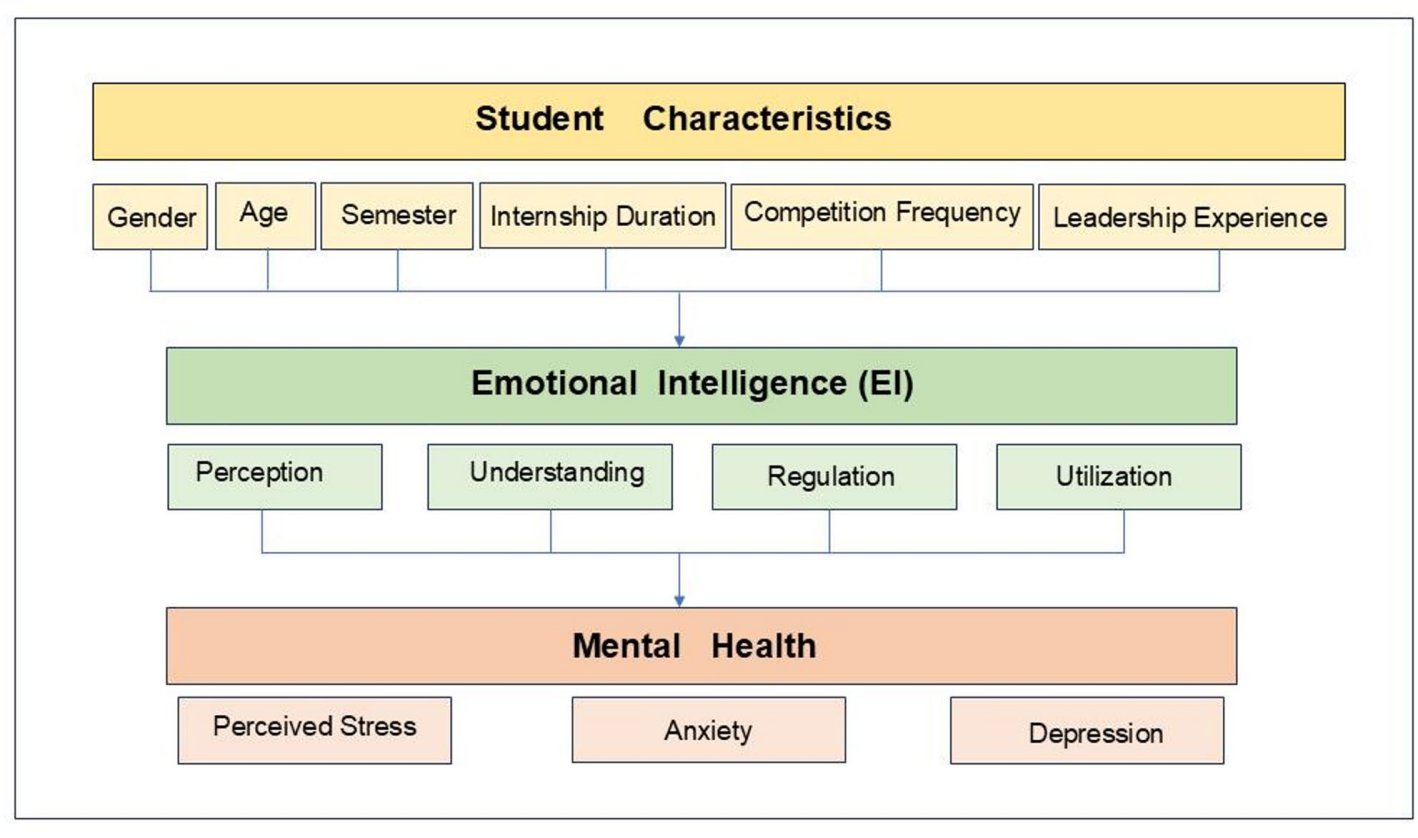
Hypothetical model of emotional intelligence and mental health

Although EI and mental health are increasingly recognized as critical aspects of medical education, empirical research among Chinese medical students remains limited. Given EI’s potential role as both a protective factor and an adaptive mechanism for managing stress, clarifying its associations with perceived stress, anxiety, and depression—and exploring gender-related differences—holds significant implications for medical education and student well-being. Figure 1 presents the hypothesized model of this study. This study aims to fill this gap by exploring the relationship between EI and mental health, with particular focus on perceived stress, anxiety, and depression. The research will examine the various factors influencing EI among Chinese medical students and investigate how EI correlates with their mental well-being, especially regarding stress, anxiety, and depression. Moreover, this study seeks to investigate whether EI mediates or moderates the relationship between gender and mental health outcomes.The findings are expected to inform targeted educational strategies and psychological interventions that enhance emotional competence, promote resilience, and improve overall mental well-being in future healthcare professionals.

## Methods

### Study population and procedures

This study employed a cross-sectional design to investigate EI among Chinese medical students. The study was conducted at the Norman Bethune Health Science Center of Jilin University in October 2024. Participants were undergraduate medical students from the 1 st, 3rd, 5th, 7th, and 9th semesters, representing a range of academic stages in their medical training. To ensure a diverse and representative sample, the survey was distributed via the Wenjuanxing platform, an online survey tool widely used in China. The recruitment process aimed to reach all students in the specified semesters, with efforts made to minimize selection bias.

The present study was conducted in accordance with the Declaration of Helsinki and approved by the Ethics Committee of the China-Japan Union Hospital, Norman Bethune Health Science Center, Jilin University. Participants were informed that their involvement was voluntary, anonymous, and confidential. Informed consent was obtained electronically through the online platform, and participants were provided with detailed information about the purpose, content, and procedures of the study before completing the survey. Upon completing the questionnaire, participants were eligible to receive a small random monetary incentive. Those who agreed to take part in the study accessed and submitted the questionnaire via the secure online platform Wenjuanxing (https://www.wjx.cn/).

Of the collected questionnaires, responses completed in less than 120 s or exhibiting logical inconsistencies (such as patterned or contradictory answers) were excluded from the dataset. After data cleaning, The final sample of 1,376 students provided data on key demographic variables, including age, gender, hospital internship duration, frequency of competitive activities, and leadership experience. These variables were collected to offer a comprehensive overview of the participants and to explore factors potentially influencing EI.

### Measures

Several validated scales were employed to assess EI and its relationship with.

psychological health among Chinese medical students. These included the Trait Emotional Intelligence Questionnaire-Short Form (TEIQue-SF), the Personal Stress and Trauma Reaction (PSTR) Stress Scale, the Self-Rating Anxiety Scale (SAS), and the Self-Rating Depression Scale (SDS).

### Trait emotional intelligence Questionnaire-Short form (TEIQue-SF)

The TEIQue-SF was developed by Petrides and Furnham to measure trait emotional intelligence [27]. The Chinese version was translated and validated by local researchers for use among Chinese populations. This instrument comprises 30 items rated on a 7-point Likert scale (1 = “completely disagree” to 7 = “completely agree”), covering four dimensions: Well-being, Self-control, Emotionality, and Sociability. Fifteen items are reverse-scored, and the total EI score is obtained by averaging all item scores, with higher scores indicating greater emotional intelligence. In the present study, the TEIQue-SF demonstrated excellent internal consistency (α = 0.93).

### Personal Stress and Trauma Reaction (PSTR) stress scale

The PSTR Stress Scale, developed by Edwards, J. R. and adapted into Chinese, measures individuals’ perceived stress responses to daily and academic challenges. It consists of 50 items rated on a 5-point Likert scale (0 = “not at all” to 4 = “extremely”). Total scores range from 0 to 200, 65 and over suggests clinically significant symptoms. This scale has shown satisfactory reliability and validity in previous studies among Chinese medical student populations. In this study, the PSTR Stress Scale showed exceptionally high reliability (α = 0.96).

### Self-Rating Anxiety Scale (SAS)

The SAS was developed by Zung [28] to evaluate the frequency and severity of anxiety symptoms. It includes 20 items, each rated on a 4-point scale (1 = “rarely” to 4 = “almost always”). Scores range from 20 to 80, 50 and over suggests clinically significant symptoms. Five items are reverse-scored. The SAS is a widely accepted and reliable tool for assessing anxiety. In the present study, the SAS demonstrated good internal consistency (α = 0.83).

### Self-Rating Depression Scale (SDS)

The SDS, also developed by Zung [29], measures the severity of depressive symptoms. It includes 20 items rated on a 4-point Likert scale (1 = “not at all” to 4 = “very much”), with total scores ranging from 20 to 80, and 50 and over suggests clinically significant symptoms. Ten items are reverse-scored. Higher scores represent greater levels of depressive symptoms. The SDS demonstrates excellent internal consistency and test-retest reliability, as well as strong content and criterion validity. In the present study, the SDS exhibited good internal consistency (α = 0.88).

### Statistical analyses

All statistical analyses were performed using R software (version 4.2.3). Descriptive statistics (means, standard deviations, and frequencies) were used to summarize sociodemographic characteristics and psychological measures. Data normality was examined using the Kolmogorov–Smirnov test, as well as skewness and kurtosis indices. The skewness and kurtosis values of all variables were within acceptable limits for large-sample parametric analyses (|skewness| < 2, |kurtosis| < 7). EI and depression scores approximated normal distributions, while anxiety and stress showed mild positive skewness and leptokurtosis. Given the large sample size (*n* = 1376), these deviations were not expected to substantially affect the robustness of the parametric tests. Between-group differences in EI and psychological health indicators (stress, anxiety, and depression) were analyzed using independent-sample t-tests and one-way ANOVA. Associations between EI and psychological variables were examined using Pearson’s correlation analysis. Multiple logistic regression models were applied to examine whether EI independently predicted dichotomized psychological distress outcomes, adjusting for key demographic and training-related covariates. Results were expressed as adjusted odds ratios (aORs) with 95% confidence intervals (CIs).

To explore the role of gender in the association between EI and psychological outcomes, we first tested potential moderation by including gender × EI interaction terms in logistic regression models. As interaction effects were not significant, mediation analysis was then conducted to assess whether EI mediated the association between gender and psychological outcomes (mediation package). Indirect effects were estimated using nonparametric bootstrapping with 5,000 resamples, and mediation was considered significant when the 95% bootstrap CI did not include zero [30]. All statistical tests were two-tailed, and a *p*-value < 0.05 was considered statistically significant.

## Results

### Demographic characteristics and EI

Descriptive data for EI and demographic variables are presented in Table 1. A total of 1376 participants were included in the study, with a mean EI score of 4.86 ± 0.81 (95% CI: 4.82–4.90). T-tests and one-way ANOVA indicated that EI differed significantly across gender (*p* = 0.036), academic semester (*p* = 0.027), leadership experience (*p* < 0.001), and competition participation frequency (*p* = 0.0007). No significant differences were observed for age or internship duration (both *p* > 0.05).

**Table 1:** Demographic characteristics and EI

Characteristic	*N *(%)1	Mean value of global trait EI (±SD)	95% CI for mean value of Global EI	*p*-value
All	1376(100%)	4.86±0.81	4.82–4.90.82.90	
Gender				0.0364
Male	645 (47%)	4.81 ± 0.837	4.75 - 4.88	
Female	731 (53%)	4.91 ± 0.791	4.85 - 4.96	
Age				0.3730
≤ 18 years old	444 (32%)	4.90 ± 0.811	4.82 - 4.97	
19 years old	282 (20%)	4.89 ± 0.810	4.79 - 4.98	
20–21 years old	464 (34%)	4.81 ± 0.821	4.73 - 4.88	
> 21 years old	186 (14%)	4.86 ± 0.807	4.75 - 4.98	
Semester				0.0273
1st	476 (35%)	4.90 ± 0.826	4.83 - 4.98	
3rd	296 (22%)	4.87 ± 0.762	4.78 - 4.95	
5th	211 (15%)	4.70 ± 0.766	4.60 - 4.80	
7th	243 (18%)	4.87 ± 0.878	4.76 - 4.98	
9th	150 (11%)	4.93 ± 0.811	4.80 - 5.06	
Hospital Internship Time				0.0878
None	697 (51%)	4.87 ± 0.812	4.81 - 4.93	
Less than 6 months	579 (42%)	4.83 ± 0.811	4.76 - 4.89	
More than 6 months	100 (7.3%)	5.02 ± 0.832	4.85 - 5.18	
Competition Activity Frequency				0.0007
0 times	433 (31%)	4.78 ± 0.850	4.70 - 4.86	
1–2 times	493 (36%)	4.83 ± 0.786	4.76 - 4.89	
3 or more times	450 (33%)	4.98 ± 0.797	4.90 - 5.05	
Leadership Experience				1.531e-07
No	647 (47%)	4.74 ± 0.828	4.68 - 4.80	
Yes	729 (53%)	4.97 ± 0.786	4.91 - 5.03	
Stress				< 2.2e-16
No	641 (47%)	5.28 ± 0.768	5.22 - 5.34	
Yes	735 (53%)	4.49 ± 0.660	4.45 - 4.54	
Anxiety				< 2.2e-16
No	1,256 (91%)	4.94 ± 0.778	4.90 - 4.99	
Yes	120 (8.7%)	3.99 ± 0.661	3.87 - 4.11	
Depression				< 2.2e-16
No	1,133 (82%)	5.03 ± 0.739	4.98 - 5.07	
Yes	243 (18%)	4.09 ± 0.691	4.00 - 4.18	

^1^*n* (%)

### Demographic characteristics and mental health (stress, anxiety, depression)

The distribution of stress, anxiety, and depression symptoms is presented in Table 2. Among the participants, 53% reported stress, 9% reported anxiety, and 18% reported depressive symptoms. These psychological indicators varied across several demographic factors, including age, academic year, and internship duration.

**Table 2:** Demographic characteristics and psychological health (stress, anxiety, depression)

				Stress			Anxiety			Depression		
Characteristic	*N* ^1^	Overall *N* = 1,376^2^	No *N* = 641^2^		Yes *N* = 735^2^	*p*-value^3^	No *N* = 1,256^2^	Yes *N* = 120^2^	*p*-value^3^	No *N* = 1,133^2^	Yes *N* = 243^2^	*p*-value^3^
All	1,376	1,376(100%)	641(47%)		735(53%)		1256(91%)	120(9%)		1133(82%)	243(18%)	
Gender	1,376					> 0.9			0.024			0.004
Male		645 (47%)	300 (47%)		345 (47%)		577 (46%)	68 (57%)		511 (45%)	134 (55%)	
Female		731 (53%)	341 (53%)		390 (53%)		679 (54%)	52 (43%)		622 (55%)	109 (45%)	
Age	1,376					0.020			0.064			0.003
≤ 18 years old		444 (32%)	192 (30%)		252 (34%)		408 (32%)	36 (30%)		375 (33%)	69 (28%)	
19 years old		282 (20%)	138 (22%)		144 (20%)		267 (21%)	15 (13%)		248 (22%)	34 (14%)	
20–21 years old		464 (34%)	207 (32%)		257 (35%)		415 (33%)	49 (41%)		363 (32%)	101 (42%)	
> 21 years old		186 (14%)	104 (16%)		82 (11%)		166 (13%)	20 (17%)		147 (13%)	39 (16%)	
Semester	1,376					0.008			0.002			< 0.001
1st		476 (35%)	206 (32%)		270 (37%)		435 (35%)	41 (34%)		403 (36%)	73 (30%)	
3rd		296 (22%)	144 (22%)		152 (21%)		284 (23%)	12 (10%)		259 (23%)	37 (15%)	
5th		211 (15%)	84 (13%)		127 (17%)		186 (15%)	25 (21%)		154 (14%)	57 (23%)	
7th		243 (18%)	122 (19%)		121 (16%)		211 (17%)	32 (27%)		193 (17%)	50 (21%)	
9th		150 (11%)	85 (13%)		65 (8.8%)		140 (11%)	10 (8.3%)		124 (11%)	26 (11%)	
Hospital Internship Time	1,376					0.004			> 0.9			0.034
None		697 (51%)	299 (47%)		398 (54%)		638 (51%)	59 (49%)		585 (52%)	112 (46%)	
Less than 6 months		579 (42%)	283 (44%)		296 (40%)		527 (42%)	52 (43%)		460 (41%)	119 (49%)	
More than 6 months		100 (7.3%)	59 (9.2%)		41 (5.6%)		91 (7.2%)	9 (7.5%)		88 (7.8%)	12 (4.9%)	
Competition Activity Frequency	1,376					0.3			0.7			0.7
0 times		433 (31%)	190 (30%)		243 (33%)		391 (31%)	42 (35%)		351 (31%)	82 (34%)	
1–2 times		493 (36%)	232 (36%)		261 (36%)		452 (36%)	41 (34%)		410 (36%)	83 (34%)	
3 or more times		450 (33%)	219 (34%)		231 (31%)		413 (33%)	37 (31%)		372 (33%)	78 (32%)	
Leadership Experience	1,376					0.5			0.3			0.13
No		647 (47%)	295 (46%)		352 (48%)		585 (47%)	62 (52%)		522 (46%)	125 (51%)	
Yes		729 (53%)	346 (54%)		383 (52%)		671 (53%)	58 (48%)		611 (54%)	118 (49%)	
EI	1,376	4.862± (0.814)	5.283± (0.768)		4.494± (0.660)	< 0.001	4.945± (0.778)	3.994± (0.661)	< 0.001	5.028± (0.739)	4.089± (0.691)	< 0.001

^1^*N* Non-missing

^2^*n* (%); Mean± (SD)

^3^Pearson’s Chi-squared test; Wilcoxon rank sum test

### Stress

Stress levels significantly differed by age (*p* = 0.020), academic year (*p* = 0.008), and internship duration (*p* = 0.004). Higher stress was observed in students aged 20–21 and those in earlier academic years. Students without internship experience reported the highest stress levels, whereas those with more than six months of internship experience reported the lowest levels.

### Anxiety

Anxiety showed significant variation by gender (*p* = 0.024) and academic year (*p* = 0.002). Males reported a higher incidence of anxiety (57%) compared to females (43%). Anxiety was more prevalent among younger students and those without internship experience.

### Depression

Depressive symptoms differed significantly by gender (*p* = 0.004), age (*p* = 0.003), academic year (*p* < 0.001), and internship duration (*p* = 0.034). Higher depression prevalence was observed in males, younger students, and those without internship experience.

### The relationship between EI and mental health (stress, anxiety, depression)

EI scores differed significantly between students with and without psychological symptoms (Table 2). Students with stress, anxiety, or depressive symptoms consistently showed lower EI scores compared with their counterparts (all *p* < 0.001). These findings suggest that lower EI is associated with a higher likelihood of psychological distress.

Multivariate logistic regression analysis further demonstrated that higher EI was independently associated with reduced odds of stress (OR 0.2, 95% CI 0.164–0.243), anxiety (OR 0.141, 95% CI 0.096–0.2), and depression (OR 0.117, 95% CI 0.085–0.158) in adjusted models (*p* < 2e-16 for all). Detailed results are shown in Table 3.

**Table 3:** Logistic regression analysis of EIand psychological health

	Medel 1		Model 2		
	OR(95%CI)	*P*-value		OR(95%CI)	*P*-value
Stress	0.214 (0.176, 0.257)	< 2e-16		0.2 (0.164, 0.243)	< 2e-16
Anxiety	0.149 (0.104, 0.208)	< 2e-16		0.141 (0.096, 0.2)	< 2e-16
Depression	0.122 (0.09, 0.163)	< 2e-16		0.117 (0.085, 0.158)	< 2e-16

*OR *odds ratio, *CI *confidence interval

Model 1:unadjusted

Model 2: adjusted for confounding factors including sex, age, semester, hospital internship time, and others

### Gender differences: interaction and mediation analyses

**Fig. 2:**
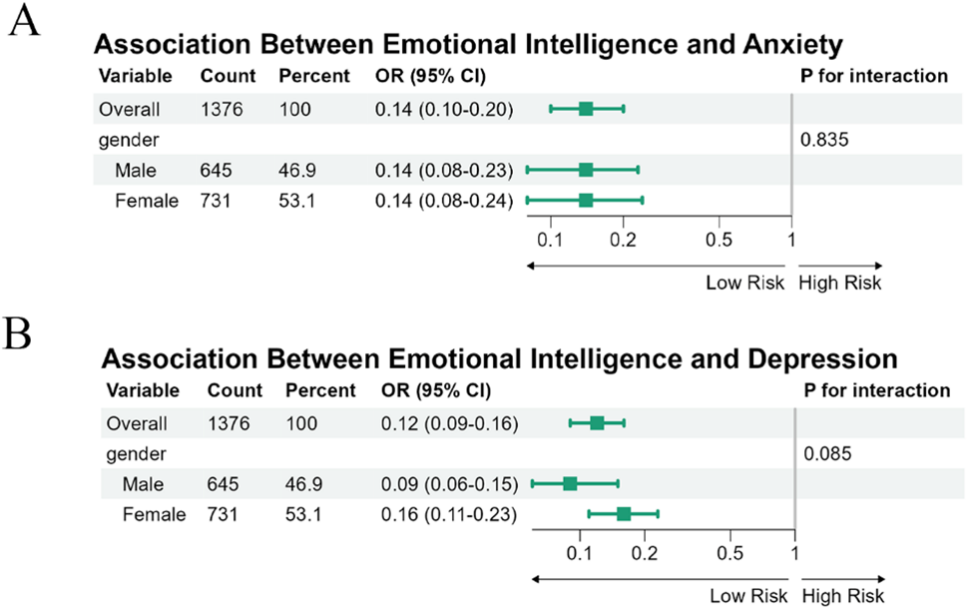
Subgroup analysis of the association between emotional intelligence (EI) and mental health outcomes (anxiety and depression) by gender. (**A**) EI and anxiety; (**B**) EI and depression.

To further clarify the role of gender in the relationship between EI and psychological distress, we conducted both interaction and mediation analyses. Interaction tests were first performed to determine whether gender moderated the association between EI and mental health outcomes. The gender × EI interaction term was non-significant for both anxiety (*P* for interaction = 0.835) and depression (*P* for interaction = 0.071), indicating that the strength of the association between EI and psychological outcomes was comparable in male and female students. These findings suggested that gender did not operate as a moderator in this context. Forest plots for the subgroup analyses are presented in Fig. 2.

**Fig. 3:**
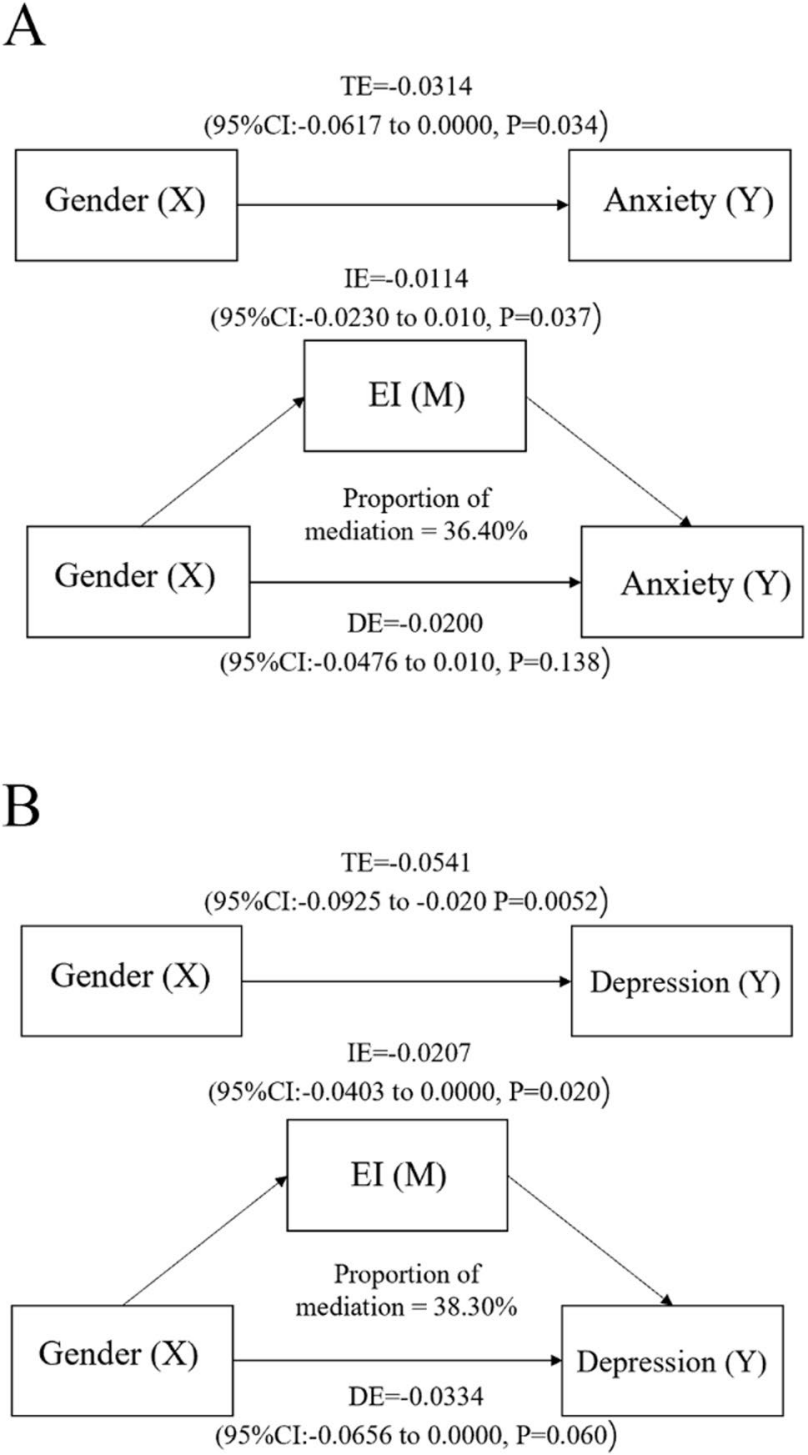
Mediation analysis of EI in the relationship between gender, anxiety, and depression (**A**) The mediation analysis of gender on anxiety. **B** The mediation analysis of gender on depression.TE: Total Effect IE: Indirect Effect DE: Direct Effect

**Fig. 4:**
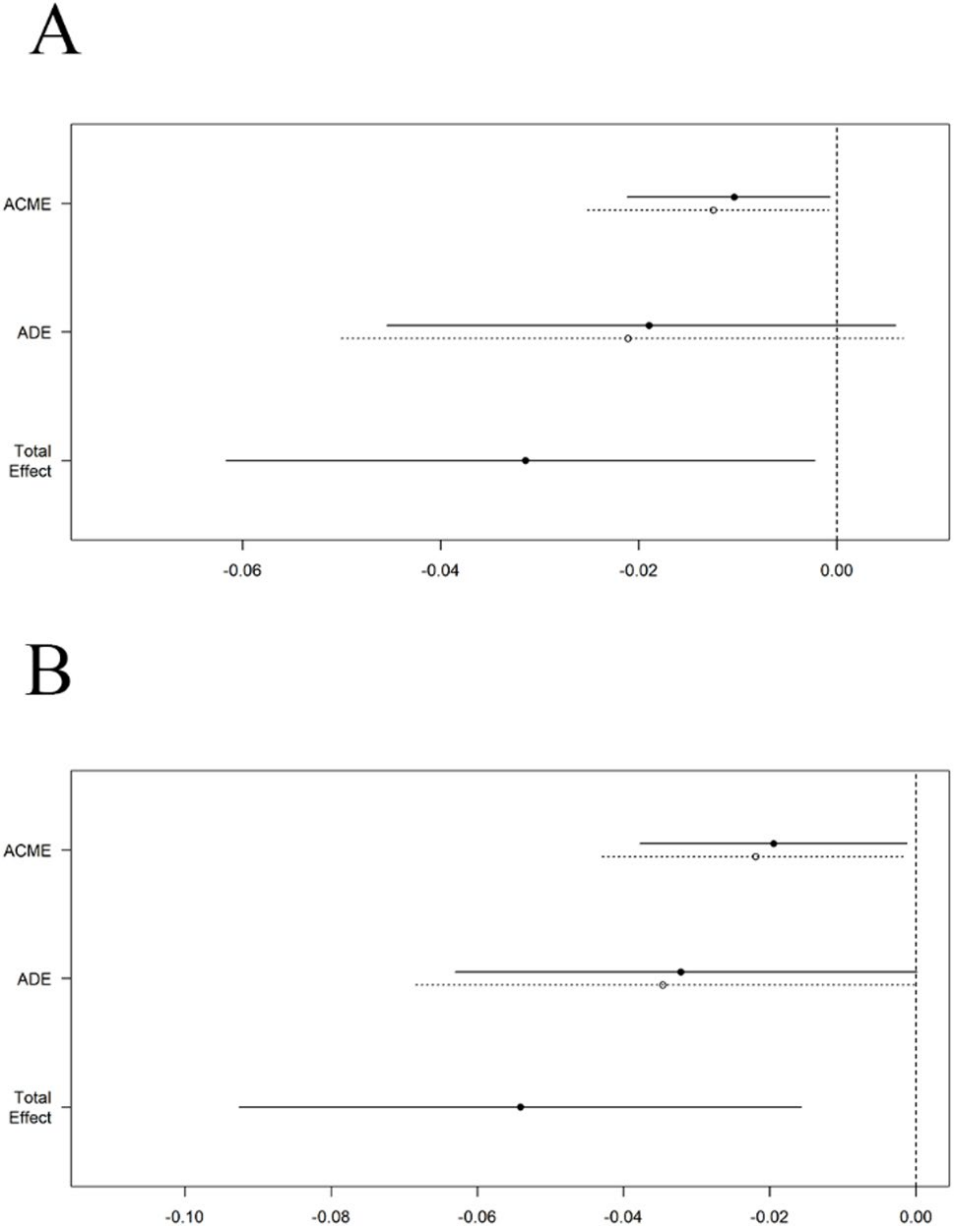
Mediation analysis of gender on anxiety and depression (ACME and ADE)(**A**) Mediation analysis of gender on anxiety. **B** Mediation analysis of gender on depression.ACME: Average Causal Mediation Effect ADE: Average Direct Effect

Given the absence of moderation, mediation analyses were performed to evaluate whether EI accounted for gender differences in psychological distress. EI partially mediated the associations between gender and both anxiety and depression (Figs. 3 and 4). The indirect effect of gender on anxiety through EI accounted for 36.40% of the total effect, while EI explained 38.30% of the association between gender and depression. After adjusting for EI, the direct effects of gender on both outcomes were attenuated and became non-significant or only marginally significant, consistent with partial mediation.

## Discussion

The primary objective of this study was to examine the determinants of EI among Chinese medical students and to clarify its association with stress, anxiety, and depression. Our hypotheses predicted that EI would vary across key demographic characteristics and that higher EI would be linked to better mental health outcomes. The results largely supported these expectations, and the mediation analysis further confirmed that EI partially explains gender differences in anxiety and depression.

### EI and its influencing factors among Chinese medical students

This study is one of the largest to investigate EI in a medical student population in China, involving a total of 1,376 participants, and the findings extend previous evidence regarding demographic variations in EI. Consistent with most prior studies, female students demonstrated higher EI than males, which aligns with research indicating stronger emotional awareness and interpersonal sensitivity among women [21–23]. However, some earlier studies have reported negligible gender differences [20, 31], suggesting that gender-related variations may be context dependent, influenced by cultural, educational, and social factors.

Our finding that EI did not differ significantly by age contrasts with reports suggesting that EI increases gradually with experience until early adulthood [20]. This divergence may reflect the unique academic trajectory of Chinese medical students, who undergo intensive standardized preparation prior to university entrance, potentially leading to earlier stabilization of emotional competencies [32]. Future longitudinal research is needed to verify this developmental pattern.

Significant differences in EI across academic years were observed, with fifth-year students showing the highest scores. This finding aligns with previous studies suggesting that clinical internships and related activities contribute to the development of EI [18, 33, 34]. However, EI did not show a progressive increase across academic years, which could be partially attributed to the impact of the COVID-19 pandemic, during this period, medical students had limited opportunities for in-person learning and clinical practice, potentially affecting the natural progression of EI development. Although the COVID-19 pandemic may have influenced opportunities for clinical practice in certain cohorts, definitive conclusions require targeted longitudinal evaluation.

Consistent with previous research, our study also found that leadership experiences and participation in competitive activities were both strongly associated with higher EI [35–37]. These findings echo earlier research suggesting that experiential learning environments enhance emotional regulation, communication, and teamwork skills. These results highlight the value of incorporating structured leadership and competition-based activities into medical training programs as potential pathways for cultivating EI.

By contrast, no significant association was observed between internship duration and EI. This inconsistency with previous literature [38] may reflect variations in clinical exposure quality or timing. Further studies with more precise documentation of clinical learning experiences are warranted.

### The relationship between EI and mental health in medical students

Consistent with international evidence, we found that medical students exhibited high levels of stress and notable rates of anxiety and depression, supporting the view that medical training places substantial psychological demands on students [18]. The results indicated that a substantial proportion of students (53%) experienced high levels of stress, while the prevalence of anxiety and depression was lower, at 9% and 18%, respectively. The associations between mental health outcomes and demographic factors such as gender, age, and academic year suggest that students encounter different stressors across stages of training, ranging from academic burden in early years to career uncertainty during later stages [17].

EI demonstrated a robust protective association with all mental health indicators. Students with higher EI reported significantly lower levels of stress, anxiety, and depression, reinforcing the conceptualization of EI as a psychosocial resource that facilitates adaptive coping, emotional regulation, and effective interpersonal functioning. As observed in previous studies, higher EI was associated with lower levels of stress, anxiety, and depression [12, 39, 40]. These findings underscore the potential value of incorporating EI training into medical curricula as a strategy to enhance students’ psychological resilience.

### The mediating role of EI in gender differences in mental health

A key contribution of this study is the demonstration that EI partially mediates gender differences in anxiety and depression. The research findings suggest that male medical students experience higher levels of anxiety and depression compared to their female counterparts. While most previous studies have indicated that females are more prone to anxiety and depression, there is a relative lack of research specifically focusing on Chinese medical students [18, 26]. Additionally, selection bias during the sampling process may also impact the study’s findings. Men and women tend to differ in their emotional recognition, expression, and management [26]. Women generally perform better in emotional expression and empathy, while men may differ in their emotional regulation and coping strategies [41, 42]. These gender differences likely contribute to variations in mental health outcomes, with EI levels partially explaining the impact of gender on mental health [43]. Higher EI helps individuals better manage their emotions, reducing the accumulation of negative emotions and thereby lowering the risk of anxiety and depression.

The mediation analysis indicates that EI accounts for a meaningful proportion of the gender differences in anxiety and depression. Students with higher EI to regulate negative emotions, adopt adaptive coping strategies, and mitigate stress responses, which may reduce their susceptibility to anxiety and depression. Although Interaction analyses indicated that gender did not moderate the association between EI and psychological outcomes, EI through which gender differences in mental health emerged.

Therefore, our study emphasizes the importance of fostering EI in mental health interventions, particularly with regard to gender-specific applications. By enhancing emotional management skills, it is possible to mitigate the impact of gender differences on mental health, helping both men and women more effectively cope with anxiety, depression, and other negative psychological states, ultimately promoting overall mental well-being.

### Practical implications

This study provides several implications for medical education. First, EI appears to be an important psychological competency linked to academic success, clinical performance, and well-being. Integrating EI training into competency-based curricula may help strengthen students’ resilience and interpersonal skills. Second, leadership and competitive activities showed strong associations with EI, suggesting that experiential, student-centered learning environments should be further promoted. Third, given the mediating role of EI in gender-related mental health differences, tailored EI-enhancing interventions may serve as an effective component of student support services. Overall, embedding EI development within medical education could contribute to cultivating more emotionally competent, psychologically healthy future physicians.

### Limitations

This study has several limitations that should be taken into account when interpreting the findings. First, the reliance on self-reported data may introduce biases, such as social desirability and recall errors, which could impact the accuracy of the results. Second, the study was conducted primarily at the Norman Bethune College of Medicine, Jilin University, which may limit the generalizability of the findings to the broader population of medical students in China. Third, the cross-sectional design of the study restricts the ability to explore changes over time in EI and mental health among medical students. Future research should employ multi-institutional sampling, longitudinal designs, and mixed-methods approaches to better clarify the mechanisms linking EI and mental health and to explore intervention strategies that may enhance EI among medical students.

## Conclusion

This study demonstrates that EI is an important psychological attribute among Chinese medical students and is closely linked to their mental health. Higher EI was consistently associated with lower levels of stress, anxiety, and depression, and EI partially mediated gender differences in psychological outcomes. These findings highlight EI as a modifiable competency with meaningful implications for medical education. Integrating EI-enhancing strategies—such as structured leadership experiences, experiential learning opportunities, and targeted emotional skills training—may strengthen students’ resilience and better equip them for the interpersonal and emotional demands of clinical practice.

Future research should employ longitudinal and multi-institutional designs to examine the developmental trajectory of EI, evaluate the effectiveness of EI-focused interventions, and further explore how EI can be integrated into competency-based medical curricula to promote both academic success and mental well-being.

## Data Availability

The data utilized in this study are available upon reasonable request from the corresponding author, with appropriate justification.

## Abbreviations

EI: Emotional intelligence
TEIQue-SF: Trait Emotional Intelligence Questionnaire-Short Form
PSTR: Personal stress and trauma reaction scale
SAS: Self-Rating Anxiety Scale
SDS: Self-Rating Depression Scale
